# 

**Published:** 1856-06

**Authors:** 


### CONTENTS OF NO. III, VOL. III.

---

### ORIGINAL COMMUNICATIONS.

Page.
Hemorrhage.................... 161
Amputation–By Asa Morgan M. D., 166

### DEPARTMENT OF SELECTIONS.

Hœmostatic Agents.............. 168
Animal Odor—Van Allen........ 170
Periodicity and Intermittency of Disease—Livezy M. D......... 177
Rheumatism—Maxson M. D...... 182
Infantile Paralysis—Adams...... 184
Treatment of Sciatica—Blackiston, 188
Bronzed Skin.................. 189
Sam Hahneman................. 192
Cataleptic Hysteria............ 195
Puerperal Peritonitis........... 199
Page.
Treatment of Croup............. 206
Meeting of the American Medical Association.................. 209
Hancock Medical Association.... 223
Iowa State Medical Society...... 224

### EDITORIAL DEPARTMENT.

Editorial....................... 225
Medical Literature.............. 229
Clergymen and Physicians....... 232
Iowa University................ 234
To Medical Men............... 235
Stomatis Materna............... 236
To Correspondents.............. 237
Matrimonial.................... 238
Monographs.................... 240

